# Staples versus sutures for skin closure in hip arthroplasty: a meta-analysis and systematic review

**DOI:** 10.1186/s13018-021-02870-z

**Published:** 2021-12-24

**Authors:** Zirui Liu, Binfeng Liu, Hao Yang, Liang Zhao

**Affiliations:** 1 The First Affiliated Hospital of Zhengzhou University, Zhengzhou, 450003 Henan China; 2 Department of Orthopaedics, The Second Xiangya Hospital, Central South University, Changsha, 410011 Hunan China

**Keywords:** Wound closure, Staples, Suture, Hip arthroplasty

## Abstract

**Objective:**

The purpose of the present study was to estimate complications and other outcomes associated with staple and suture closure after hip arthroplasty through meta-analysis techniques and a systematic review.

**Methods:**

We searched for articles in EMBASE, PubMed, Medline, Web of Science and the Cochrane Library. To determine the eligibility of the searched trials, Cochrane Collaboration's Review Manager software was used to perform the meta-analysis.

**Results:**

Five randomized controlled trials and one retrospective cohort trial were included in our study. Our study indicated that for skin closure after hip arthroplasty, the risks of superficial infection and prolonged discharge were higher with staples than with sutures. There was no significant difference between the two groups in terms of allergic reaction, dehiscence, inflammation, abscess formation, the Hollander Wound Evaluation Scale or patient's satisfaction with skin closure methods. However, suturing required a longer operating time.

**Conclusions:**

Closure with sutures is associated with lower risks of superficial infection and prolonged discharge than closure with staples following hip arthroplasty, but it may take more time.

## Introduction

Hip arthroplasty is the final treatment for degenerative and traumatic hip disease. With the ageing of the population and the progress of medical technology, the rate of arthroplasty has consistently been rising over the past decades [1, 2]. Hip arthroplasty includes total hip arthroplasty and hemiarthroplasty [3]. It is estimated that by 2030, major total hip arthroplasty operations in the USA will be performed 572,000 times a year, an increase of 174% since 2005 [4].

Wound complications are one of the main morbidities of hip arthroplasty and can prolong hospitalization duration or lead to readmission, increase costs and reduce patient satisfaction [5, 6]. Surgical site infections (SSIs) are one of the most common and important complications after hip arthroplasty. Patients with early incision infections after hip arthroplasty have poor clinical scores in terms of postoperative pain and function. Some incision infections may spread inward, leading to deep infections and failure of the prosthesis [7]. Good skin closure in hip arthroplasty can achieve appropriate closure and rapid healing, with acceptable cosmesis and minimal complications such as infection, delayed dehiscence and haematoma[5]; in addition, the stress of early activities and accelerated wound rehabilitation programmes highlight the importance of skin closure [8].

Many materials have been used for skin closure in hip arthroplasty, among which sutures and staples are the most common [9]. However, there seems to be no consensus in the literature on which method of skin closure is superior in hip arthroplasty. Some studies show that the incidence of complications between skin staples and sutures is similar; however, studies also report that sutures can reduce costs and that sutures are less painful to remove [6, 10–12]. In addition, Yao Lu et al. found that the incidence of complications with suture closure was significantly lower than that with staple closure [13]. The main disadvantage of sutures is that it takes a longer time to suture a wound, and sutures require surgeons to have better suture skills [14]. Unlike sutures, the surface of the staples rarely touches the edge of the wound, and staples do not penetrate to the depth of the incision. Staplers do much less damage to wound defence and reduce the immune response. In the process of wound closure and removal, staples are also considered faster than sutures [15, 16]. However, Singh et al. [6] showed that using staples was more than three times costlier than using sutures. Therefore, further critical evidence is needed to prove which skin closure method is more suitable for surgical wound closure after hip arthroplasty.

Until recently, some meta-analyses and systematic reviews comparing staples with sutures for skin closure in orthopaedic procedures or total knee arthroplasty have been published [17–20]. However, after extensive searching through a large number of studies in the past, we found no related study comparing the wound complications, perioperative details and resource utilization of patients whose wound was closed with staples or sutures after hip arthroplasty. Meanwhile, some randomized controlled trials comparing staples and sutures for wound closure in hip arthroplasty have been published in recent years. The purpose of the present study was to estimate complications and other outcomes associated with staple and suture closure after hip arthroplasty through meta-analysis and systematic review. We hypothesized that skin staples would be associated with better outcomes in the assessment of all relevant variables.

## Materials and methods

### Literature search

Two investigators (ZL, BL) independently conducted an extensive search of electronic databases such as EMBASE, PubMed, Medline, Web of Science and the Cochrane Library on studies published between 1996 and October 2021. When searching, we used the following keywords and their combinations: staples, clips, suture, arthroplasty, hip, wound, closure, and skin. Our study included only English-language publications on human trials. In addition, the bibliographies of the included studies and dissertations were searched for additional sources. A manual search of relevant trials, reviews and related articles was also performed. Authors were contacted, when possible, to obtain missing information.

### Inclusion and exclusion criteria

To be included in this study, the trials had to meet the following inclusion criteria: (1) be full-text randomized controlled trials and high-quality retrospective cohort studies; (2) compare staples versus sutures; (3) include patients treated with primary hip arthroplasty, including total hip arthroplasty or hemiarthroplasty; (4) include at least one of the key outcomes. The exclusion criteria were as follows: (1) studies on skin adhesives used for skin closure; (2) studies on barbed sutures; (3) studies were case reports, discourses, basic research, conference papers, non-English articles and other studies that did not contain results to our interest; (4) study data that could not be extracted. How deeper tissues were closed and the type of suture or stitch technique used were not considered exclusion criteria. The two authors independently assessed whether each article met the criteria for inclusion and then discussed their differences until they reached a consensus.

### Selection of the literature

After eliminating duplicates, two independent researchers (ZL, BL) scanned the titles and abstracts according to predetermined selection criteria and selected randomized controlled trials that might be relevant. The researchers retrieved hard copies of all relevant articles and read the full texts for further identification. Relevant data were extracted through a predetermined standardized procedure involving the first author, the year of publication, the demographic characteristics of countries, the participants and the treatment options for each group.

### Quality assessment

The Cochrane collaboration tool for assessing risk of bias was used to evaluate the methodological quality of the included randomized controlled trials [21]. This tool focuses on the internal validity of the trial and assessment of risk for possible bias in different phases of the trial. The details are as follows: random sequence generation, allocation concealment, blinding of outcome assessment, blinding of participants and personnel, incomplete outcome data, selective reporting, and other bias. Each item was classified according to a high, low, or unclear risk of bias that is represented as high (H), low (L), and unclear (U), respectively. The Newcastle–Ottawa Scale (NOS) was used to assess the quality of cohorts and case–control studies [22]. NOS ranges from 0 to 9 stars; research scores above 5 are considered high quality. All the assessments were conducted by the two independent reviewers (ZL, BL).

### Data extraction

The relevant data from the eligible papers were subjected to double extraction by two authors (ZL, HY) according to a predefined standardized protocol. We extracted the baseline of each included study from trials that included the following information: design, closure material, age (mean and range), sex, BMI, closure length, stitch technique for suture, cohort size, follow-up, and information on the study objective. When inadequate information existed in the studies, contacting the study authors to obtain and clarify the relevant data was essential, as specified by the standardized protocol.

### Outcome assessment

We extracted data on superficial infection, deep infection, prolonged discharge, abscess, wound dehiscence, allergic reaction, and inflammation as our primary evaluated outcomes. The secondary outcomes evaluated included wound closure time, length of stay in the hospital, the Hollander Wound Evaluation Scale (HWES), and the visual analogue scale (VAS).

### Statistical analysis

The Review Manager Software Package (RevMan Version 5.3, The Cochrane Collaboration, Copenhagen, 2014) was used to generate forest plots. The overall effect of staples or sutures on wound closure was calculated as the weighted average of the inverse variance adjusted individual effects and 95% confidence interval (95% CI). The statistical heterogeneity among the individual studies was evaluated based on the Cochrane Q test and the *I*^2^ index [23], and statistical heterogeneity was confirmed if *I*^2^ was > 50% and *P* < 0.10 [24]. A variance-based fixed effect model was applied to calculate the pooled effect; otherwise, a random effect model was used in the presence of statistically significant heterogeneity [25]. If appropriate, the heterogeneity was identified and explained using a subgroup analysis [23]. Evidence grading was evaluated according to the Grading of Recommendations, Assessment, Development and Evaluation system [26].

### Ethical statement

As all analyses were grounded on previously published studies, ethical approval was not necessary.

## Results

### Literature search

**Fig. 1:**
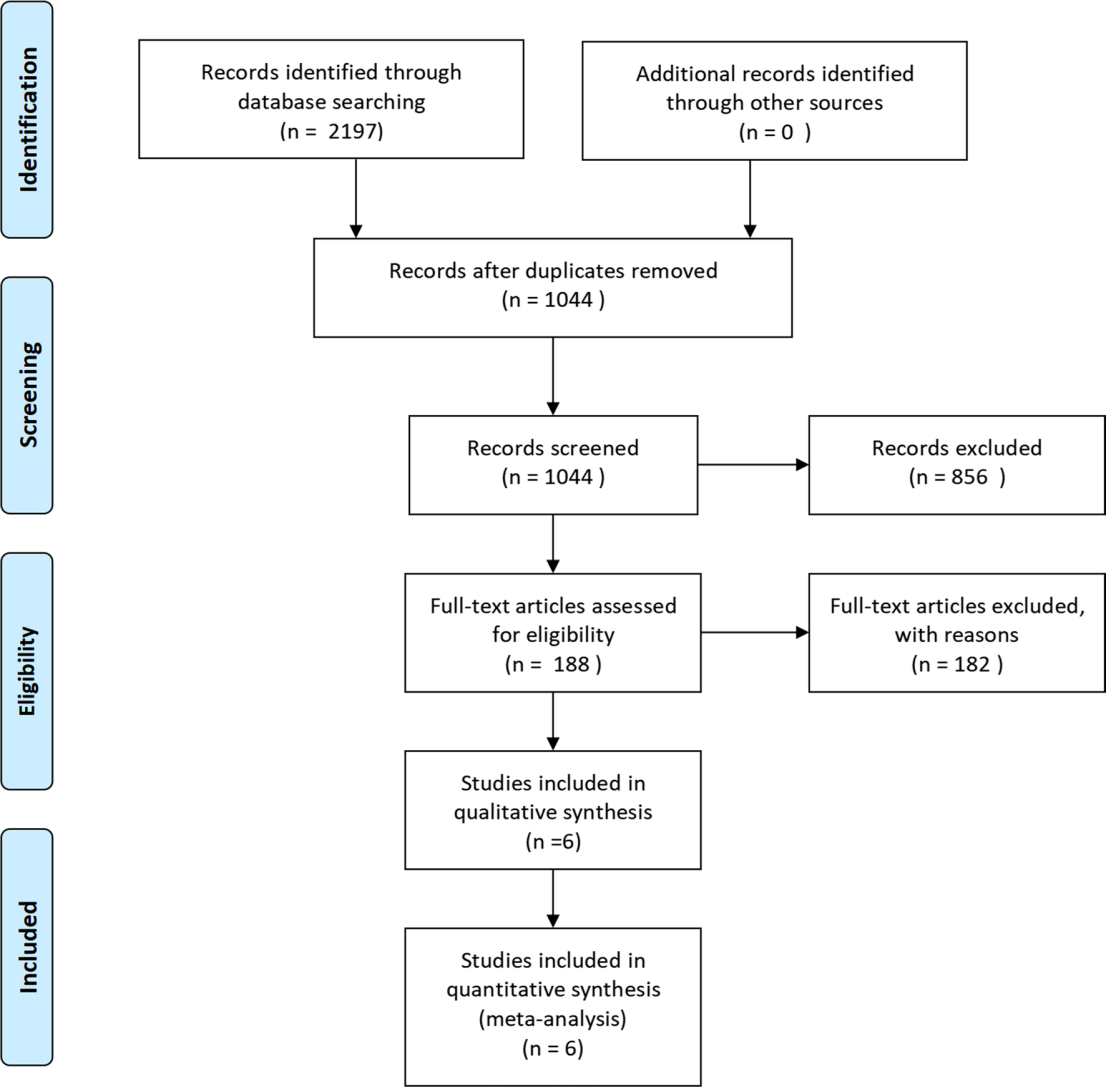
Flow diagram of the studies included

There were 2197 articles in the initial literature search. According to the inclusion and exclusion criteria, 188 articles were retained after removing duplicates and screening the title and abstract. Finally, after careful reading of the full text, five randomized controlled trials [6, 11, 12, 27, 28] and one retrospective cohort trial [13] were included in our study. The flowchart of the studies included is presented in Fig. 1.

### Characteristics of the trials

A total of 1286 patients, 627 in the staples group and 659 in the sutures group were included in our study. Every patient we selected from this eligible study had undergone total hip replacement or hemiarthroplasty. One RCT[27] was divided into three groups: the skin clips group, OCA (2-octylcyanoacrylate) group, and sutures group. The OCA group was excluded from this meta-analysis. The retrospective cohort trial [13] was also divided into three groups: the subcuticular sutures group, the staples group and the interrupted sutures group. Because the subcuticular sutures group used staples as a supplementary means of skin closure, we also removed the subcuticular sutures group from this study. The mean age of the participants ranged from 55.7 years to 71 years in the staples group and from 57.8 years to 70 years in the sutures group. The sample sizes of the studies included ranged from 17 to 268. More detailed baseline characteristics of the eligible studies are shown in Table 1.

**Table 1:** The characteristics of included studies

Author/year	Period	Study	Operation	Closure material	Stitch technique for suture group	Size		Age (mean)		SEX (male)		BMI			Closure length			Follow-up(days)
						Staples	Sutures	Staples	Sutures	Staples	Sutures	Staples	Sutures		Staples	Sutures		
Khan/2006	2004	RCT	THA	3.0 poliglecaprone Suture, skin staples	Subcutaneous continuous suture	36	33	71 (33–78)	69 (49–88)	20/36	17/33	26.9 (21.1–38.9)	27.7 (19.8–38.1)		10.5 (7.5–22)	10 (7.5–15)		56–84
Singh/2006	2001–2002	RCT	THA, hemiarthroplasty	Subcuticular vicryl skin clips	Subcutaneous continuous suture	17	17	Unclear		Unclear		Unclear			Unclear			2,5,7,10,14
Buttaro/2015	2011–2012	RCT	THA	3.0 polypropylene suture, staples	Intradermal suture	112	119	62 (range 21–91)		Unclear		Unclear			12.2 (9–22)			15,45
Lu, Y/2018	2013–2015	RC	THA, hemiarthroplasty	Nylon sutures, staples	interrupted suture	111	141	64.1 (25–90)	67.3 (22–99)	53/111	56/141	24.4 (17.5–31.6)	23.5 (14.9–20.6)		Unclear			30
Rui, M/2018	2014–2015	RCT	THA	4.0 absorbable subcuticular suture, staples	Subcutaneous continuous suture	83	82	55.7 (33–78)	57.8 (35–79)	36/83	39/82	27.1 (17.6–33.4)	26.8 (16.4–32.2)		13.8 (12–16)	13.6 (12–15)		90,365
Mallee/2020	2012–2016	RCT	THA	Absorbable suture or Donati-stitches, staples	Intradermal suture/interrupted suture	268	267	70 (29–92)	70 (38–90)	182/268	175/267	28(18–48)	27(17–46)		Unclear			14,90,180,365

The characteristics of all included studies

RC: retrospective cohort; RCT: randomized controlled trial; THA: total hip arthroplasty

### Risk of bias assessment

**Fig. 2:**
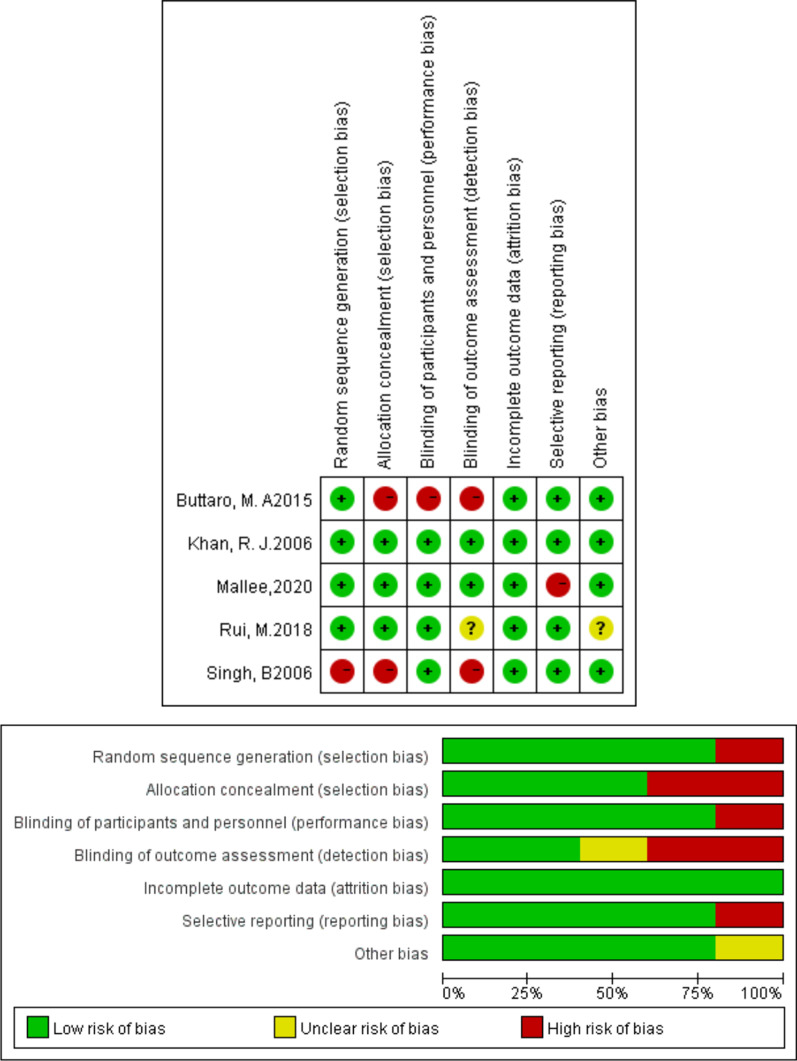
Risk of bias summary and graph of randomized controlled trials

Based on the Cochrane Collaboration recommendation, four RCTs [11, 12, 27, 28] reported the detailed methods of random sequence generation. Two RCTs [12, 27] reported allocation concealments. The participants and personnel were blinded in 3 RCTs [6, 12, 27]. In addition, full details of withdrawals and dropouts were described in all studies. In some studies, the follow-up rate was 100%. Details of deviation risk are shown in Fig. 2. The bias risk of the retrospective cohort trial assessed with NOS is shown in Table 2.

**Table2:** Risk of bias assessment of retrospective cohort trial

Risk of bias assessment	Lu et al. (2018)
Selection	3
Comparability	2
Outcome/exposure	2
Total score	7

The Newcastle–Ottawa scale (NOS) was used to assess the quality of nonrandomized studies with its design, content and ease of use directed to the task of incorporating the quality assessments in the interpretation of meta-analytic results

### Superficial infection

**Fig. 3:**
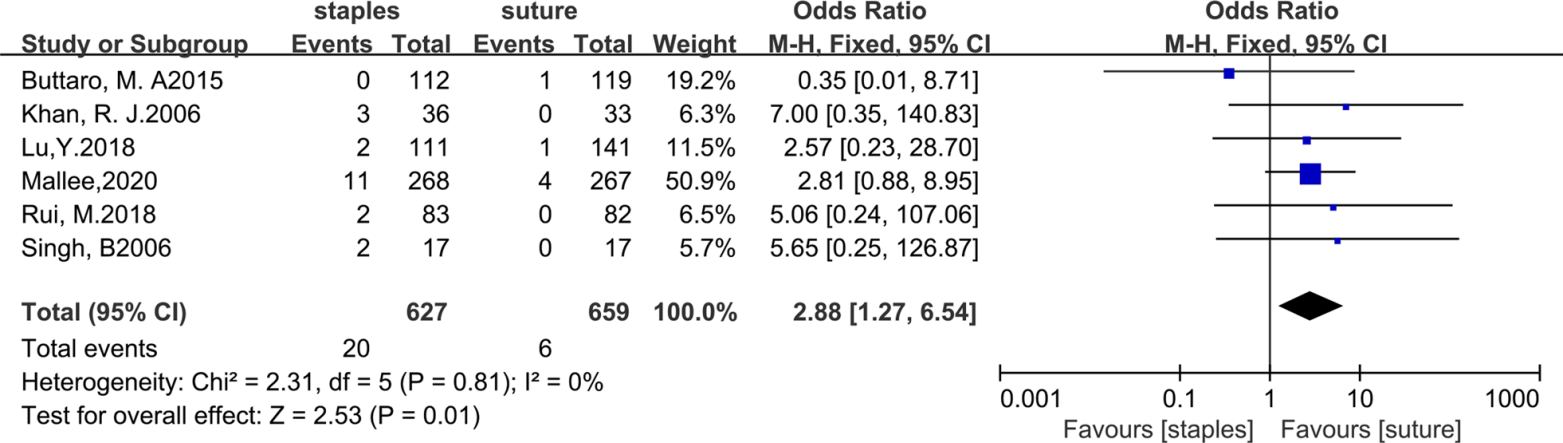
Forest plot for superficial infection rate after hip arthroplasty

**Fig. 4:**
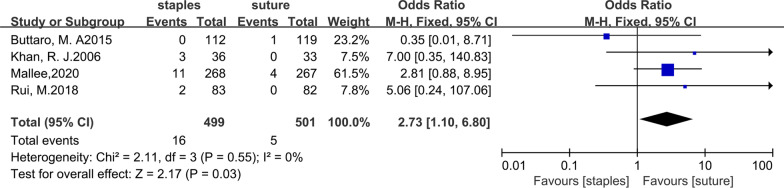
Forest plot for superficial infection rate after total hip arthroplasty

All six trials included in our study compared the superficial infection rates between the staples group and the suture group. Twenty out of 627 patients in the staples group were infected, while 6 out of 659 patients in the sutures group were infected. The fixed-effects meta-analysis of the 6 trials showed that the risk of superficial infection was higher with staples than with sutures for skin closure after hip arthroplasty. The odds ratio of superficial infection was 2.88 (95% CI 1.27–6.54; *P* = 0.01), and there was no heterogeneity (*χ*^2^ = 2.31; *I*^2^ = 0%; *P* = 0.81). The forest plots are illustrated in Fig. 3. In addition, we further compared the superficial infection rate between staples and sutures for skin closure after total hip arthroplasty. Data regarding superficial infection after total hip arthroplasty were reported in four studies[11, 12, 27, 28]. After total hip arthroplasty, 16 out of 499 patients in the staples group were infected, while 5 out of 501 patients in the sutures group were infected. The fixed-effects meta-analysis showed that the risk of superficial infection was higher with staples than with sutures for skin closure after total hip arthroplasty, with an OR (odds ratio) of 2.73 (95% CI 1.10 to 6.80; p = 0.03), and there was no heterogeneity (χ^2^ = 2.11; I^2^ = 0%; P = 0.55). The forest plots are illustrated in Fig. 4. All pooled outcomes comparing staples to sutures for skin closure after arthroplasty are listed in Table 3.

**Table3:** Pooled outcomes comparing staples to suture for skin closure after arthroplasty

Outcomes	No. of studies	No. of case		OR (95% CI)	*P* value	Heterogeneity	
		Staples	Suture			*I*^2^ (%)	*P* value
Superficial infection	6	20/627	6/659	2.88 (1.27,6.54)	0.01*	0	0.81
Deep infection	3	7/491	4/527	1.70 (0.56,5.21)	0.35	0	0.43
Superficial infection(THA)	4	16/499	5/501	2.73 (1.10,6.80)	0.03*	0	0.55
Deep infection(THA)	2	5/380	4/386	1.24 (0.35,4.35)	0.74	0	0.74
Prolonged discharge	5	55/544	21/577	2.88 (1.72,4.83)	< 0.0001*	0	0.83
Abscess	2	0/147	1/174	0.30 (0.01,7.54)	0.46	Not applicable	
Wound dehiscence	3	4/462	2/490	0.42 (0.02,10.41)	0.60	Not applicable	
Allergic reaction	2	2/148	6/152	0.39 (0.09,1.69)	0.21	0	0.86
Inflammation	2	12/128	7/158	3.78 (0.05,317.93)	0.56	87	0.006

THA: total hip arthroplasty, OR: odds ratio; the outcome of inflammation was calculated using the Mantel–Haenszel random-effects model. Others outcome were calculated using the Mantel–Haenszel fixed-effects model

*P value < 0.05

## Deep Infection

**Fig. 5:**
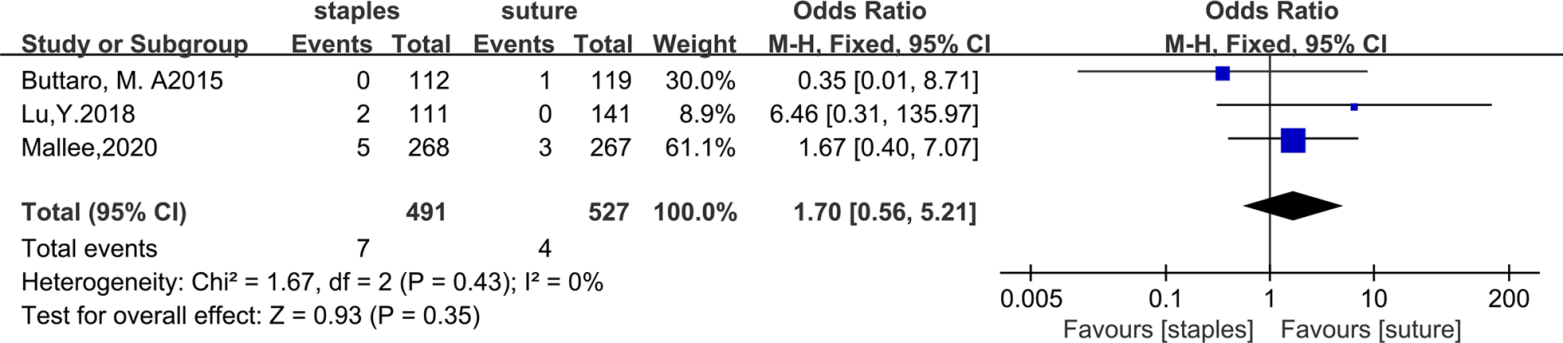
Forest plot for deep infection rate after hip arthroplasty

**Fig. 6:**
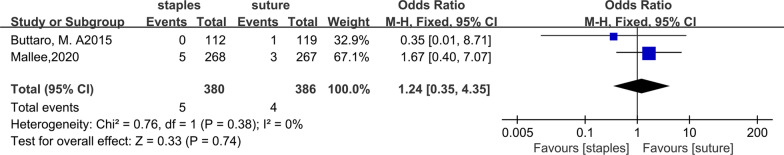
Forest plot for deep infection rate after total hip arthroplasty

Three of the trials compared the deep infection rates between the staples group and the sutures group. Seven out of 491 patients in the staples group were infected, while 4 out of 527 patients in the sutures group were infected. The fixed-effects meta-analysis of the 6 trials showed that the risk of deep infection was higher with staples than with sutures for skin closure after hip arthroplasty. The odds ratio of deep infection was 1.70 (95% CI 0.56–5.21; *P* = 0.35), and there was no heterogeneity (*χ*^2^ = 1.67; *I*^2^ = 0%; *P* = 0.43). The forest plots are illustrated in Fig. 5. Similarly, we further compared the deep infection rates between staples and sutures for skin closure after total hip arthroplasty. Data regarding deep infection after total hip arthroplasty were reported in four studies [11, 12, 27, 28]. After total hip arthroplasty, 5 out of 380 patients in the staples group were infected, while 4 out of 386 patients were infected in the sutures group. The fixed-effects meta-analysis showed that the risk of deep infection was higher with staples than with sutures for skin closure after total hip arthroplasty, with an OR (odds ratio) of 1.24 (95% CI 0.35–4.35; *P* = 0.74) and no heterogeneity (*χ*^2^ = 0.76; *I*^2^ = 0%; *P* = 0.38). The forest plot is illustrated in Fig. 6.

### Prolonged discharge

**Fig. 7:**
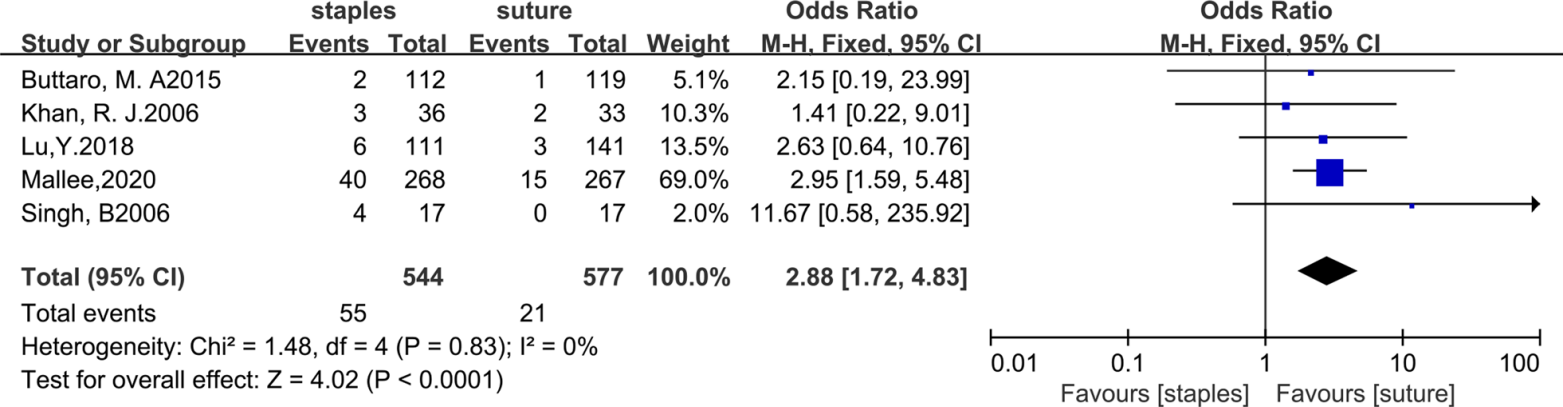
Forest plot for prolonged discharge

Overall, 5 studies [6, 11, 13, 27, 28] reported on discharge after hip arthroplasty. Wound discharge lasting at least 4 days was recorded as ‘prolonged discharge’ in these studies; 55 out of 544 patients in the staples groups had prolonged discharge, while 21 out of 577 patients in the sutures groups had prolonged discharge. The fixed-effects meta-analysis of all 5 trials revealed that the risk of prolonged discharge was lower with sutures than with staples for skin closure after hip arthroplasty. The odds ratio of prolonged discharge was 2.88 (95% CI, 1.72 to 4.83; P < 0.0001), and there was no heterogeneity (*χ*^2^ = 1.48; *I*^2^ = 0%; *P* = 0.83). The forest plot is listed in Fig. 7.

### Abscess

**Fig. 8:**
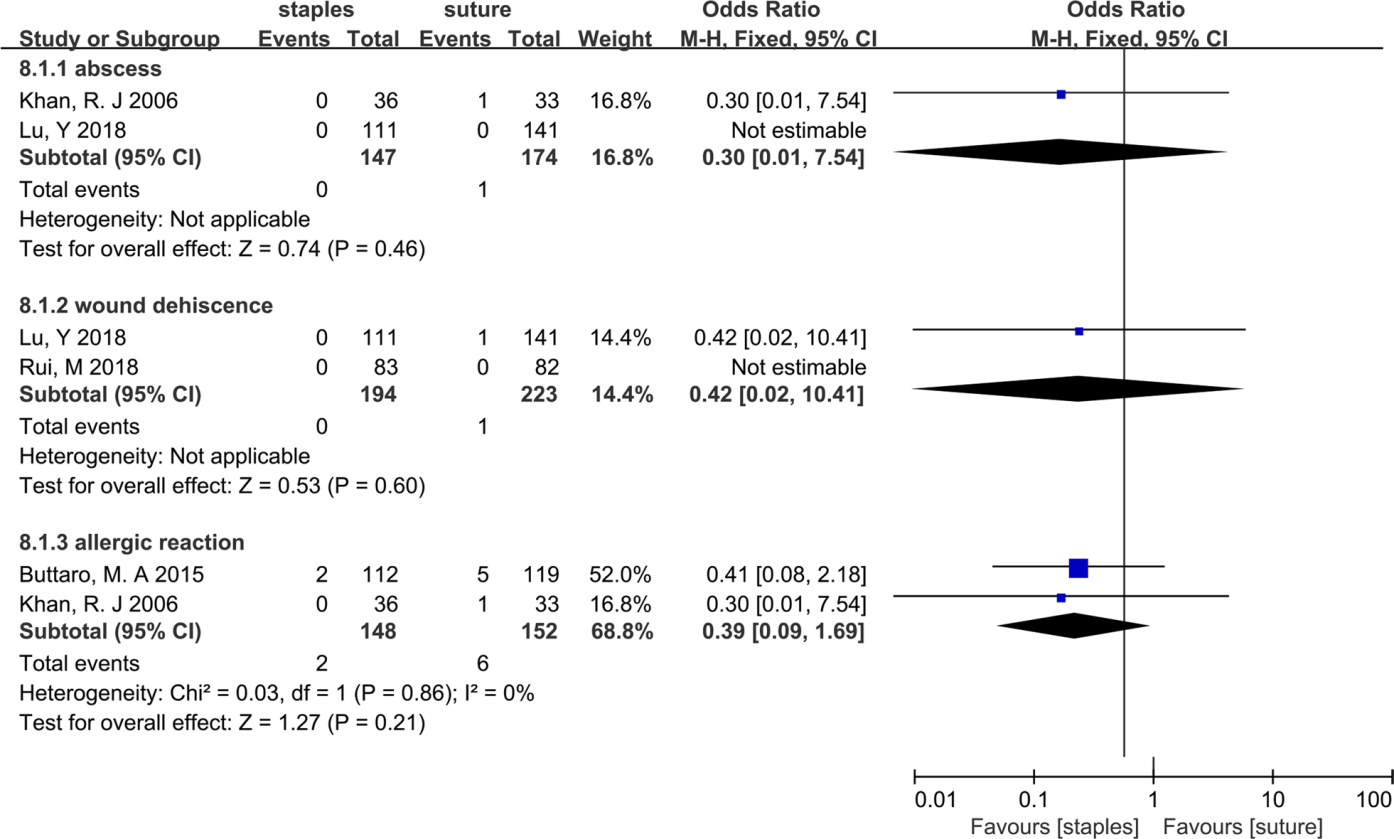
Forest plot for abscess, wound dehiscence, allergic reaction

Two studies [13, 27] compared the number of participants who developed wound abscesses after hip arthroplasty. From a total of 321 patients, only one patient from the sutures group developed an abscess. The OR-based models revealed that the incidence of abscesses was similar between skin staples and sutures (OR = 0.30; 95% CI 0.01–7.54; *P* = 0.46), with no heterogeneity. The forest plots are illustrated in Fig. 8.

### Wound dehiscence

Three studies [12, 13, 28] reported data on wound dehiscence. Four out of 462 patients in the staples group experienced wound dehiscence, while 2 out of 499 patients in the sutures group experienced wound dehiscence. The fixed-effects meta-analysis of 3 trials revealed that there was no significant difference in the risk of wound dehiscence between the two groups after hip arthroplasty (OR = 0.42; 95% CI 0.02–10.41; *P* = 0.60), with no heterogeneity. The forest plots are illustrated in Fig. 8.

### Allergic reaction

Two trials [11, 27] reported allergic reactions. Two out of 300 patients had allergic reactions, and our findings showed that there was no significant difference in the risk of allergic reaction between the staples groups and the sutures groups after hip arthroplasty (OR = 0.39; 95% CI 0.09–1.69; *P* = 0.21; heterogeneity: *χ*^2^ = 0.03, *P* = 0.86, I^2^ = 0%). The forest plots are illustrated in Fig. 8.

### Inflammation

**Fig. 9:**
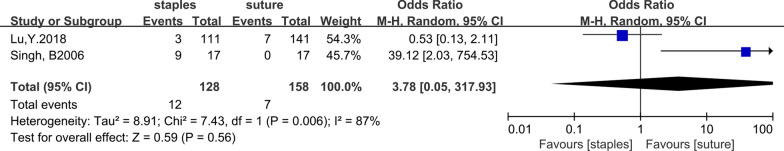
Forest plot for inflammation

A total of two trials [6, 13] reported inflammation. In the staples groups, there were signs of inflammation in 12 out of 128 participants, while in the sutures groups there were only seven out of 158 participants with signs of inflammation. However, our pooled result revealed that the differences noted between the staples group and sutures group were not statistically significant (OR = 3.78; 95% CI 0.05–317.93; *P* = 0.56; heterogeneity: *χ*^2^ = 7.43, *P* = 0.006, *I*^2^ = 87%). Because the heterogeneity was more than 50%, we chose the fixed-effects model. In addition, there are no additional data to support our further sensitivity or subgroup analysis. The forest plots are illustrated in Fig. 9.

### Systematic review

In addition to the six outcomes assessed in our study, four other secondary outcomes (length of stay, Holland Wound Evaluation Score, closure time, and visual analogue score) were compared in the systematic review section of this study. The included trials did not provide appropriate data types to conduct a meta-analysis.

#### Wound closure time

Two studies reported the time required for wound closure. Khan et al. [27] and Rui et al. [12] both showed that wound closure with staples was significantly faster than with sutures. The data on wound closure time with staples or sutures are listed in Table 4.

**Table 4:** Closure time, LOS, HWES, VAS

Outcomes	Author/year	Staples		Suture		*P* value
		Median	Interquartile range	Median	Interquartile range	
Closure time(s)						
	Khan/2006	30	18–30	150	210	NA
	Rui, M/2018	24.7	21.3–29.4	357.7	332.1–383.1	p < 0.001
LOS(day)						
	Khan/2006	4	3–6	4	4–6	NA
	Lu, Y/2018*	16.9	8–30(range)	17.3	9–35(range)	NA
	Rui, M/2018	12	11–13	6.0	5–8	p < 0.001
HWES						
	Khan/2006	5.3	5–6	6	5–6	NA
	Rui, M/2018	4	4–5	5	4–5	0.170
VAS**						
	Khan/2006	95	88–100	94	86–99	NA
	Rui, M/2018	6	6–8	7	6–8	0.180

NA: not applicable; LOS: length of stay; HWES: Hollander Wound Evaluation score; VAS: Visual Analogue Scale

^*^The study of Lu et al. provides only the median and range of length of stay, not interquartile range. **The study of Khan et al. judged that patient satisfaction with skin closure technology was assessed with the Visual Analogue Scale (VAS) between 0 and 100, of which 100 expressed the greatest satisfaction, while Rui et al. assessed with the Visual Analogue Scale (VAS) between 0 and 10 expressed the greatest satisfaction, of which 10 indicated the greatest satisfaction

#### Length of stay

Three studies indicated the length of stay in the hospital for wound closure with skin staples or sutures after hip arthroplasty. Khan et al. [27] and Lu et al. [13] both showed that there were no significant differences in the median length of stay in the hospital. However, Rui et al. [12] reported the length of stay in the hospital in the staples group compared with the sutures group. The median and interquartile ranges of length of stay in the hospital are listed in Table 4.

#### HWES (Hollander Wound Evaluation Score)

The HWES is a scoring rule to evaluate surgical wounds and predict wound healing. The HWES evaluates the wound for steps, irregular contour, edge separation, edge inversion, excessive deformation and overall appearance. Each category is graded with a score of 0 or 1. The total score is 0–6, and wounds with a score of 6 have the best prognosis. Two studies used the Hollander Wound Evaluation Score to evaluate wounds after hip arthroplasty. In the two studies [12, 27], the HWES of the staples groups was lower than that of the sutures groups, but there was no significant difference in Holland's wound evaluation score. The Hollander Wound Evaluation Scores are listed in Table 4.

#### VAS (visual analogue scale)

Two studies [12, 27] used the visual analogue scale (VAS) to evaluate the satisfaction of patients according to skin wound closure methods. The study by Khan et al. judged patient satisfaction with skin closure technology with a VAS score between 0 and 100, of which 100 denotes the greatest satisfaction, while Rui, M et al. assessed patient satisfaction with a VAS score between 0 and 10, with 10 denoting the greatest satisfaction. Both of these studies reported that there was no significant difference between the satisfaction of patients in the staples group and those in the sutures group after hip arthroplasty. The Visual Analogue Scale (VAS) scores are listed in Table 4.

## Discussion

Hip arthroplasty is the final treatment for debilitating hip joint disease. In recent years, the number of hip replacements has been increasing. [2]. The main purpose of skin closure in hip arthroplasty is to obtain sufficient closure, to promote rapid healing with an acceptable appearance, and to reduce complications such as delayed healing, dehiscence, haematoma and infection [10, 29]. There are many kinds of skin closure materials used in hip arthroplasty, and staples and sutures have served as primary materials for superficial skin closure [30]. At present, there are no clear clinical guidelines for skin closure after hip arthroplasty. Overall, our goal of this systematic review and meta-analysis was to provide valuable information about sutures versus staples for wound closure in hip arthroplasty. In the present study, we evaluated superficial infection, deep infection, prolonged discharge, abscess, wound dehiscence, allergic reaction, inflammation, wound closure time, length of stay in the hospital, Hollander Wound Evaluation Score, and patient satisfaction with skin closure methods from five eligible RCTs and one retrospective cohort trial that compared outcomes with staples or sutures for skin wound closure after hip arthroplasty.

In the present study, we found that the risk of superficial infection with sutures was less than twice that with staples. We found that our pooled result was similar to that of a previous meta-analysis by Smith et al. [19], who reported that after orthopaedic surgery, the risk of wound infection was significantly higher when the wound was closed with staples than with sutures, especially in patients who underwent hip surgery. Shetty et al. [31] also showed that the incidence of superficial wound infection was significantly higher when metal staples were used to suture skin wounds after hip fractures. In addition, our meta-analysis showed that the risk of superficial infection with staples was higher than that with sutures for skin closure after total hip arthroplasty. However, regardless of hip arthroplasty or total hip arthroplasty, there was no significant difference between the staples group and the sutures group in the comparison of deep infection which may be related to the use of antibiotics and deep tissue sutures. Surgical site infection increases the burden on the health care system, increases the length of stay, rehospitalization rate and cost of health care, and adversely affects the quality of life and function of patients [32]. Therefore, our meta-analysis results may be helpful to practitioners in making the choice between staples and sutures for wound closure following hip arthroplasty. However, there are still some potential prognostic factors that remain uncertain. For example, surgical techniques, expertise, aseptic techniques, antibiotic time and patient-specific prognostic factors may also influence surgical site infection risk [33]. Therefore, we need more high-quality randomized, blind, and long-lasting follow-up RCTs to be published and subsequently included in our analysis to help us choose the best wound closure method following hip arthroplasty. In the meantime, our findings suggest that the prolonged wound discharge rate was significantly lower in the sutures group. Our results and those of previous studies show that skin wounds closed with sutures are more resistant to infection, which then affects wound healing and wound discharge [34]. However, many potential factors affect the use of sutures as well, such as the accuracy aligning dermal margins and the doctor's skill, which may also affect the exudation of wound discharge [35].

In addition, we compared the incidence of abscess, wound dehiscence, allergic reaction and inflammation between the two groups, and our analysis showed that there were no significant differences in the outcomes between the staples group and sutures group. Our results lend support to the conclusions of Krishnan et al. [18], who reported no significant difference in allergic reaction, inflammation, dehiscence, or abscess formation between the two groups after orthopaedic surgery. However, it is important to note that only two RCTs analysed LOS (length of stay), allergic reaction, inflammation, dehiscence, and abscess formation between the two groups. Finally, our systematic review results show that there was no significant difference in HWES and patient satisfaction with skin wound closure methods after hip arthroplasty. Therefore, this is not a factor that affects our choice of skin closure methods after hip arthroplasty. Khan et al. [27] and Lu et al. [13] both showed that there were no significant differences in the median length of stay in the hospital. However, Rui et al. [12] reported that the median (interquartile range) length of hospital stay (days) in the staples groups was 12 (11–13) days versus 6.0 (5–8) days in the sutures group. The results suggest that the length of hospitalization following staple closure may be slightly longer than that following suture closure. Khan et al. [27] reported an estimated two-minute savings in time using staples for skin closure. Rui, M[12] also reported that the use of staples for skin closure compared with sutures can save approximately five minutes. The results suggest that the use of staples may be slightly faster than the use of skin sutures after hip arthroplasty.

Although this analysis was performed on 6 studies, and these different studies compared outcomes with different methods, we tried our best to analyse the data that could be included. The limitations of this meta-analysis and systematic review include the small number of included high-quality RCTs, unified surgical management programmes and rehabilitation programmes as well as publishing language. Some studies or guides have documented the distorting effects of location bias and publication bias on system evaluation and meta-analysis [36–38]. The poor quality of evidence in original studies reduces the quality of evidence in meta-analysis. Because of some characteristics of surgical techniques, orthopaedic surgeons cannot carry out blinded studies. Therefore, attention should be given to interpreting the conclusions of this meta-analysis. Due to the small number of studies included in this meta-analysis, we expect more high-quality RCTs to be published in the future, and future research should focus on high-quality randomized controlled trials. Detailed baseline characteristics and detailed patient recruitment flowcharts should be provided.

## Conclusion

In summary, our meta-analyses and systematic review suggest that the risks of superficial infection and prolonged discharge are higher with staples than with sutures for skin closure after hip arthroplasty. However, there was no significant difference in allergic reaction, inflammation, dehiscence, abscess formation, the Hollander Wound Evaluation Score or satisfaction among patients who received either wound closure technique after hip arthroplasty. However, the suture technique may require a longer operating time.

## Data Availability

All data and material generated or analysed during this study are included in this published article.

## Abbreviations

SSIs: Surgical site infections
RCTs: Randomized controlled trials
H: High
L: Low
U: Unclear
NOS: Newcastle–Ottawa scale
95% CIs: 95% Confidence intervals
OCA: 2-Octylcyanoacrylate
OR: Odds ratio
HWES: Hollander Wound Evaluation Score
VAS: Visual analogue scale AR Androgen receptor
LOS: Length of stay
